# Mechanisms Underlying the Protective Effect of the Peroxiredoxin-6 Are Mediated via the Protection of Astrocytes during Ischemia/Reoxygenation

**DOI:** 10.3390/ijms22168805

**Published:** 2021-08-16

**Authors:** Egor A. Turovsky, Elena G. Varlamova, Egor Y. Plotnikov

**Affiliations:** 1 Institute of Cell Biophysics of the Russian Academy of Sciences, Federal Research Center “Pushchino Scientific Center for Biological Research of the Russian Academy of Sciences”, 142290 Pushchino, Russia; 2 A.N. Belozersky Institute of Physico-Chemical Biology, Lomonosov Moscow State University, 119992 Moscow, Russia; plotnikov@belozersky.msu.ru; 3 V.I. Kulakov National Medical Research Center of Obstetrics, Gynecology and Perinatology, 117997 Moscow, Russia

**Keywords:** ischemia, apoptosis, necrosis, neuron, astrocyte, peroxiredoxin-6, cell protection, mitochondria, calcium, ROS

## Abstract

Ischemia-like conditions reflect almost the entire spectrum of events that occur during cerebral ischemia, including the induction of oxidative stress, Ca^2+^ overload, glutamate excitotoxicity, and activation of necrosis and apoptosis in brain cells. Mechanisms for the protective effects of the antioxidant enzyme peroxiredoxin-6 (Prx-6) on hippocampal cells during oxygen-glucose deprivation/reoxygenation (OGD/R) were investigated. Using the methods of fluorescence microscopy, inhibitory analysis, vitality tests and PCR, it was shown that 24-h incubation of mixed hippocampal cell cultures with Prx-6 does not affect the generation of a reversible phase of a OGD-induced rise in Ca^2+^ ions in cytosol ([Ca^2+^]_i_), but inhibits a global increase in [Ca^2+^]_i_ in astrocytes completely and in neurons by 70%. In addition, after 40 min of OGD, cell necrosis is suppressed, especially in the astrocyte population. This effect is associated with the complex action of Prx-6 on neuroglial networks. As an antioxidant, Prx-6 has a more pronounced and astrocyte-directed effect, compared to the exogenous antioxidant vitamin E (Vit E). Prx-6 inhibits ROS production in mitochondria by increasing the antioxidant capacity of cells and altering the expression of genes encoding redox status proteins. Due to the close bond between [Ca^2+^]_i_ and intracellular ROS, this effect of Prx-6 is one of its protective mechanisms. Moreover, Prx-6 effectively suppresses not only necrosis, but also apoptosis during OGD and reoxygenation. Incubation with Prx-6 leads to activation of the basic expression of genes encoding protective kinases—PI3K, CaMKII, PKC, anti-apoptotic proteins—Stat3 and Bcl-2, while inhibiting the expression of signaling kinases and factors involved in apoptosis activation—Ikk, Src, NF-κb, Caspase-3, p53, Fas, etc. This effect on the basic expression of the genome leads to the cell preconditions, which is expressed in the inhibition of caspase-3 during OGD/reoxygenation. A significant effect of Prx-6 is directed on suppression of the level of pro-inflammatory cytokine IL-1β and factor TNFα, as well as genes encoding NMDA- and kainate receptor subunits, which was established for the first time for this antioxidant enzyme. The protective effect of Prx-6 is due to its antioxidant properties, since mutant Prx-6 (mutPrx-6, Prx6-C47S) leads to polar opposite effects, contributing to oxidative stress, activation of apoptosis and cell death through receptor action on TLR4.

## 1. Introduction

Reduced blood flow (ischemia) causes rapid progression of such pathological processes such as tissue hypoxia, tissue acidification, and impaired membrane permeability, impaired activity of the plasma membrane ion channels and depletion of ATP reserves, which leads to cell damage. Restoration of blood flow (reperfusion) in tissues damaged by ischemia leads to even greater ROS (reactive oxygen species) production and increased oxidative stress [1,2]. ROS in brain cells include superoxide anion radical (O_2_^•−^), hydroperoxide radical (HO_2_^•^), hydrogen peroxide (H_2_O_2_), hydroxyl radical (HO^•^), singlet oxygen (^1^O_2_) [3]. Uncontrolled increase in ROS levels is activated in cells as a result of the altered expression level of the protective proteins in response to changes in the environment such as hypoxia and ischemia [4,5]. With oxidative stress, inflammatory processes are also activated, and the production of pro-inflammatory cytokines is increased [6]. Along with the damaging effect, moderate ROS formation performs important signaling functions in brain cells [3]. It is known that short-term hypoxia causes the phenomenon of hypoxic preconditioning and protects brain cells from damage by activating the protective genes [7,8], and long-term hypoxia leads to an increase in ROS levels promoting the activation of genes engaged in damage and suppression of protective ones [9,10]. Therefore, for the normal functioning of neuroglial networks in the brain, it is necessary to maintain a balance between generation of ROS and its utilization by antioxidant systems. The activity of the antioxidant system of the brain cells is supported by low-molecular antioxidant compounds and antioxidant enzymes of the protein nature. The former are flavonoids, various vitamins, thiols, etc., they are usually found in food or in the bioactive additives. The latter are catalase, superoxide dismutases (SOD), glutathione peroxidases, glutathione reductases, thioredoxins and peroxiredoxins which are expressed in the brain cells [11,12]. It takes a lot of energy to maintain a high level of activity of antioxidant systems in the brain and any increase in antioxidant synthesis can lead to a restriction of the level of intracellular substrates [3]. So, the utilization of toxic ROS concentrations by exogenous antioxidant enzymes is a promising way to protect the brain from damage, especially in ischemia/reoxygenation.

Peroxiredoxins (Prx) hold a special place among the compounds with antioxidant properties, since they, in addition to direct action—neutralization of ROS—are also chaperones and serve as signaling and regulatory molecules [13]. At present, six peroxiredoxins have been identified in mammals. Prx can be divided by the number of conservative cysteine residues in the active site and catalytic mechanism into typical 2- Cys (Prx1-4), atypical 2-Cys (Prx5) and 1-Cys (Prx6). Peroxiredoxins have no significant amino acid sequence homology to the other antioxidant enzymes although they can be very similar in spatial structure to thiol oxidoreductases, since they share a common structural motif named the thioredoxin fold [14]. A body of evidence shows that impaired Prx-6 expression correlates with neurodegenerative diseases. Patients with Alzheimer’s disease are characterized by reduced levels of Prx-6 expression in tissues [15]. The involvement of Prx-6 in the regulation of mitophagy was also established. The accumulation of ROS under cardiovascular risk factors leads to the accumulation of Prx-6 and PTEN-putative kinase 1 (PINK1) stabilization in damaged mitochondria. In addition, depletion of Prx-6 in the cell contributes to stabilization of PINK1, accumulation of autophagic marker p62, translocation of Parkin to mitochondria, and lipidation of microtubule-associated protein 1 light chain 3 (LC3) [16]. Prx6 inside cells are localized mainly in the cytosol, and they are accumulated less in lysosomes and mitochondria under normal conditions [16,17,18]. However, treatment of GFP-Parkin-overexpressing HeLa cells with the CCCP ionophore causes ROS formation and Prx-6 accumulation in mitochondria. Further stress of the mitochondria results in depletion of Prx-6, excessive accumulation of ROS, and even more damage to the mitochondria. Thus, Prx-6 can play a role in keeping balance for protection of mitochondria through the activation of mitophagy at a certain stage of pathology. NAC (N-acetyl cysteine) can inhibit mitophagy induced by depletion of Prx-6 activities when mitochondria are damaged, but overexpression of Prx-6 does not promote mitophagy, thereby suggesting that there are additional mechanisms associated with Prx-6 and mitophagy [16].

On the whole, antioxidant therapy has shown its effectiveness on models of neurodegeneration in vitro, while clinical trials have faced a number of difficulties, and first of all difficulties in delivery of antioxidants to the brain cells and chemical instability of the antioxidants. The advantage of Prx-6 is that it is an endogenous antioxidant enzyme of the brain. Furthermore, understanding the mechanisms of its action during ischemia/reoxygenation can contribute to the development of a new promising brain protective strategy.

## 2. Results

### 2.1. Prx-6 Protects Hippocampal Cells from Death during Ischemia by Suppressing Global [Ca^2+^]i Increase

**Figure 1:**
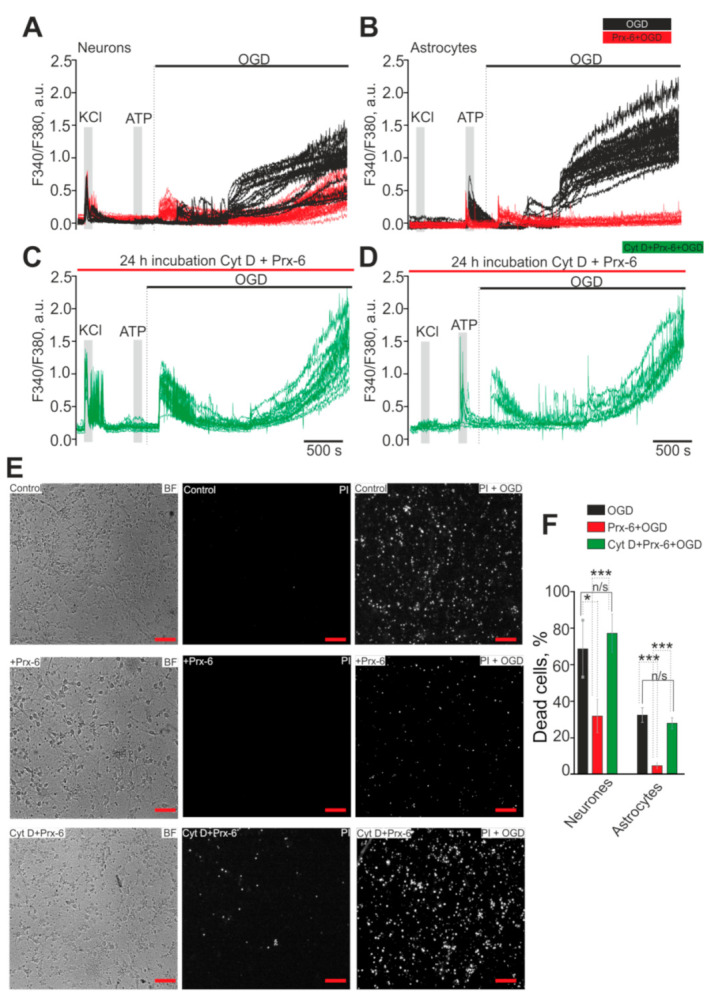
Protective effect of 24-h incubation of hippocampal cells with 100 μM of the antioxidant enzyme peroxiredoxin-6 (Prx-6) occurs through the mechanism of endocytosis. (**A**,**B**) Ca^2+^-signals of neurons and astrocytes during a 40-min OGD in control (**A**,**B**, black traces) and after 24-h incubation with Prx-6 (100 µM) (**A**,**B**, red traces). (**C**,**D**) Ca^2+^-signals of neurons (**C**) and astrocytes (**D**) during a 40-min OGD after 24-h incubation with 100 μm Prx-6 and with an endocytosis blocker, Cytochalasin D (Cyt D, 5 μM). There is an abolition of the inhibitory effect of Prx-6 on the global increase in [Ca^2+^]_i_ during OGD. Here are presented the Ca^2+^-signals of cells in single experiments obtained using the same cell culture.(**E**) Images of hippocampal cell culture in transmitted light (BF), the Propidium Iodide fluorescence detection channel before the experiment (PI) and after 40-min OGD treatment (PI + OGD). The effect of 24-h incubation of cells with Prx-6 and Prx-6 with Cyt D on cell viability after 40 min OGD. The white dots represent the PI-stained nuclei of necrotic cells. (**F**) The average number of PI-stained neurons and astrocytes that died due to OGD-induced necrosis in the absence of Prx-6 (OGD) and after 24-h incubation with 100 µM of Prx-6 (Prx-6 + OGD) or Prx-6 with 5 µM Cyt D (Cyt D + Prx-6) (% ± SE). Short-term applications of 35 mM of KCl and 10 µM of ATP were used to detect neurons and astrocytes, respectively. Statistical significance was assessed using paired *t*-test. n/s—data not significant (*p* > 0.05), * *p* < 0.05 and *** *p* < 0.001. Comparison Neurones with Astrocytes significant, *** *p*-level < 0.001. Scale bar = 30 µm.

Figure 1 shows that OGD causes generation of two-phase Ca^2+^-signals in neurons (Figure 1A) and astrocytes (Figure 1B), and the second phase of global increase in [Ca^2+^]_i_ correlates with necrotic cell death (Figure 1E, top line—PI + OGD), as evidenced by the appearance of fluorescence of the PI probe. After 40 min of OGD, necrosis is detected in 72 ± 23% of neurons and 56 ± 14% of astrocytes (Figure 1F, black columns). Before exposure to OGD in the control group, necrosis is detected only in occasional cells (Figure 1E, top line-PI). Previously, we discovered that incubation of cells with the antioxidant enzyme—Prx-6 promotes the survival of neurons through regulation of the level of [Ca^2+^]i and this protective effect rises with increasing incubation time [19]. However, the mechanisms of the protective action of Prx-6 on hippocampal cells (especially astrocytes) remained unexplored and became the focus of our current research. Incubation of hippocampal cell cultures with 100 μM of Prx-6 for 24 h results in complete suppression of the second phase of an OGD-induced increase in [Ca^2+^]_i_ in astrocytes (Figure 1B—red traces) and a 70% decrease in this parameter in neurons compared to Control (Figure 1A—black traces). In neurons, after incubation with Prx-6, a lag phase with a duration of 21 ± 6 min (5 ± 3 min in control) is also observed between the first reversible Ca^2+^-signal under OGD and global increase in [Ca^2+^]_i_, which can also be regarded as a protective effect. It is known that the global increase in [Ca^2+^]_i_ under OGD is directly associated with the death of neurons and astrocytes [20]. Incubation of cells with Prx-6 reduces the death of neurons to 36 ± 5%, and astrocytes to 8 ± 4% (Figure 1F), which indicates the most pronounced protective effect of this antioxidant enzyme on hippocampal astrocytes. In this case, the ratio of astrocytes and neurons in the culture of the hippocampus is 31 ± 16% and 68 ± 15%, respectively (Supplementary Figure S1).

The protective effect of 24-h incubation hippocampal cells with Prx-6 is provided by the mechanisms of endocytosis. Application of Prx-6 to the cell culture together with the endocytosis blocker Cytochalasin D (Cyt D, 5 μm) for 24 h leads to the abolishment of the suppression effect of OGD-induced Ca^2+^ signals in neurons (Figure 1C) and astrocytes (Figure 1C). At the same time, after 40 min of OGD death of 76 ± 24% of neurons and 27 ± 11% of astrocytes occurs (Figure 1E—CytD + Prx-6, Figure 1F).

Therefore, the antioxidant enzyme Prx-6 is able to protect the brain cells from necrotic death during ischemia (OGD) by inhibiting the phase of the global increase in [Ca^2+^]_i_. The protective effect of Prx-6 is more pronounced in astrocytes, where there is a complete suppression of the OGD-induced increase in [Ca^2+^]_i_, whereas in neurons—there is 70% suppression of this phase of the OGD-induced signal. The protective effect of Prx-6 is provided by endocytosis during 24-h incubation of cell cultures.

### 2.2. A Change in the Redox-Status of Cells and Suppression of ROS Ans NO Production Contribute to the Protective Properties of Prx-6 in the Brain

**Figure 2:**
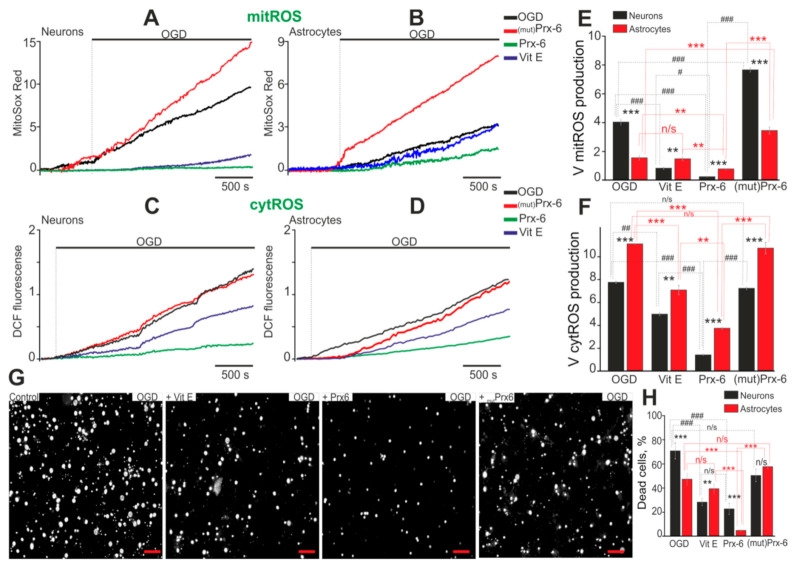
Effects of antioxidant enzyme Prx-6, mutant Prx-6 (mutPrx-6, Prx6-C47S) and natural antioxidant vitamin E (Vit E) on OGD-induced ROS production in mitochondria (mitROS), cytosol (cytROS) and cell viability. (**A**,**B**) Registration of ROS production in mitochondria of the neurons (**A**) and the astrocytes (**B**) during OGD (40 min) depending on a 24-h incubation of the cell cultures with Prx-6 (100 μM), mutPrx-6 (100 μM) and Vit E (100 μM). (**C**,**D**) Registration of ROS production in cytosol of the neurons (**C**) and the astrocytes (**D**) during OGD (40 min) depending on a 24-h incubation of the cell cultures with Prx-6 (100 μM), mutPrx-6 (100 μM) and Vit E (100 μM). Average curves plotted according to data on several dozen cells are represented. (**E**,**F**) Effect of the compounds under study on the rate of mitROS (**E**) and cytROS (**F**) production in hippocampal neurons and astrocytes. The average rates of ROS production within the interval from the point of OGD onset to the end point of the experiment are represented. (**G**,**H**) Effect of 24-h incubation with Vit E, Prx-6 and mutPrx-6 on the cell viability after 40 min OGD. Statistical significance was assessed using paired *t*-test. n/s—data not significant (*p* > 0.05), ** *p* < 0.01, and *** *p* < 0.001; # *p* < 0.05, ## *p* < 0.01, ### *p* < 0.001. Scale bar = 30 µm.

Ischemia is known to induce generation of ROS by mitochondria (mROS) [21] and cytosolic enzymes [22], and a number of antioxidants can inhibit ROS production and Ca^2+^-responses in the brain cells [23]. Hippocampal cells were simultaneously loaded with a Ca^2+^-sensitive dye such as Fura-2 and a mitochondrial ROS sensor, MitoSox-Red. Neurons were distinguished from astrocytes based on the presence of Ca^2+^-signals for a short-term depolarization with application of 35 mM KCl, and OGD was then created. Hippocampal neurons are characterized by high basic production of ROS (Figure 2A, black curve, up to OGD symbol), while in astrocytes mitochondria no pronounced ROS production is observed without exposure to OGD (Figure 2B, black curve, up to OGD symbol). Under OGD, mROS production in neurons increases rapidly (Figure 2A, black curve, OGD), when in astrocytes, on the average, a lag-period of about 4 ± 3 min is seen within the interval from the initial point of exposure to OGD and the starting point of ROS production (Figure 2B, black curve, OGD). Interestingly, the rate of OGD-induced ROS production in mitochondria of the neurons is 2.3 times higher than in astrocytes (Figure 2E, OGD). Vitamin E is an exogenous antioxidant which has shown high efficiency as a mitochondrial ROS scavenger and a neuroprotector [23]; that is why Vit E was used to compare its effects with antioxidant effects of Prx-6. The incubation of hippocampal cells using vitamin E for 24 h results in complete suppression of basic mROS production in the neurons, as well as in a 4-fold decrease of OGD-induced ROS formation when compared to that carried out under OGD conditions (Figure 2A, blue curve; Figure 2E, Vit E). At the same time, no effect of Vit E on OGD-induced mROS production in astrocytes was observed (Figure 2B, blue curve), and the rate of ROS formation did not change reliably as compared to that under OGD conditions without Vit E (Figure 2E, Vit E). However, the tests on cell survival showed that Vit E indeed protected mainly hippocampal neurons from necrosis, 31 ± 8% neuronal death was observed (75 ± 22% in the OGD group), and approximately 41 ± 9% of astrocytes died (48 ± 6% in the OGD group) (Figure 2G, +Vit E; Figure 2H).

Treatment of hippocampal cells with Prx-6 for 24 h provides not only complete suppression of basic ROS production in mitochondria of neurons, but also inhibition of OGD-induced mROS formation, thereby reducing the process rate to almost zero (Figure 2A, green curve; Figure 2E, Prx-6). In astrocytes, Prx-6 also suppresses ROS formation during OGD, but decreases the rate of mROS formation by 2-fold as compared to OGD (Figure 2B, green curve; Figure 2E, Prx-6). Prx-6 reduces the amount of necrotic neurons to 22 ± 8%, and astrocytes to 3 ± 1% (Figure 2G,H).

Numerous in vivo and in vitro studies revealed that protective Prx-6 effect on mammalian tissues exposed to various deleterious effects was seen due to the antioxidant properties [12,24]. The incubation of the cell cultures with mutant peroxiredoxin-6 (100 µM, Prx6-C47S), which is deprived of peroxidase activity and does not function as an antioxidant, has no effect on basic mROS production in the neurons, but increases ROS formation by 2-fold during OGD (Figure 2A, red curve) when compared to OGD (Figure 2E, mutPrx-6). A similar effect of an increase in mROS production was also observed in astrocytes during OGD, of the production rate increases by 1,6-fold (Figure 2B, red curve; Figure 2E, mutPrx-6). The increased mROS production affects cell survival, raising the percentage of necrotic neurons and astrocytes up to 54 ± 7% and 62 ± 9%, respectively (Figure 2G, Figure 2H, +mutPrx-6).

Cytosolic enzymes are another source of ROS in cells. To measure the production of predominantly cytosolic ROS (cytROS), cells were loaded with a DCF-DA fluorescent probe. During OGD, there was an increase in cytROS production in neurons (Figure 2C) and astrocytes (Figure 2C), but the rate of production was higher in astrocytes (Figure 2F). Vitamin E leads to suppression of cytROS production in neurons (Figure 2C) and astrocytes (Figure 2D) by 33.7% and 35.8%, respectively (Figure 2F). After incubation of cells with Prx-6, a more pronounced suppression of cytROS production during OGD occurs in neurons by 79% and astrocytes by 67% (Figure 2F). Mutant Prx-6 did not significantly reduce cytROS production in hippocampal cells during OGD (Figure 2C,D,F).

**Figure 3:**
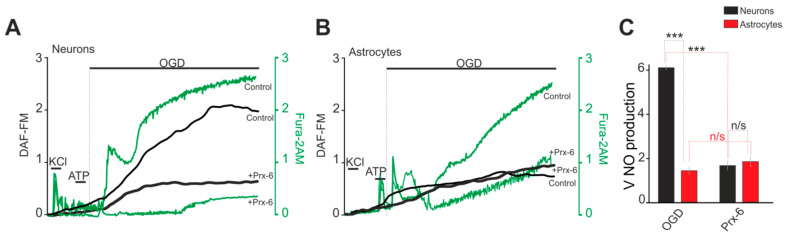
Effects of antioxidant enzyme Prx-6 on OGD-induced NO production. (**A**,**B**) Registration of NO production and Ca^2+^-responses in the neurons (**A**) and the astrocytes (**B**) during OGD (40 min) depending on a 24-h incubation of the cell cultures with Prx-6 (100 μM). Average curves plotted according to data on several dozen cells are represented. (**C**) Effect of the Prx-6 under study on the rate of NO production in hippocampal neurons and astrocytes. The average rates of NO production within the interval from the point of OGD onset to the end point of the experiment are represented. Short-term applications of 35 mM of KCl and 10 µM of ATP were used to detect neurons and astrocytes, respectively. Statistical significance was assessed using paired *t*-test. n/s—data not significant (*p* > 0.05), *** *p* < 0.001.

Enhanced nitric oxide (NO) production can contribute significantly to the toxic effects of OGD. By simultaneously measuring the dynamics of [Ca^2+^]_i_ and NO production, we were able to show that in response to OGD, there is an increase in NO production in neurons (Figure 3A) and astrocytes (Figure 3B). The phase of increased NO production correlates with the first phase of [Ca^2+^]_i_ increase during OGD, but in astrocytes the rate of NO production is almost 3 times lower than in neurons (Figure 3C). Incubation of hippocampal cells with Prx-6 leads to suppression of NO production during OGD in neurons (Figure 3A, + Prx-6, Figure 3C), but does not affect this parameter in astrocytes (Figure 3B,C).

**Figure 4:**
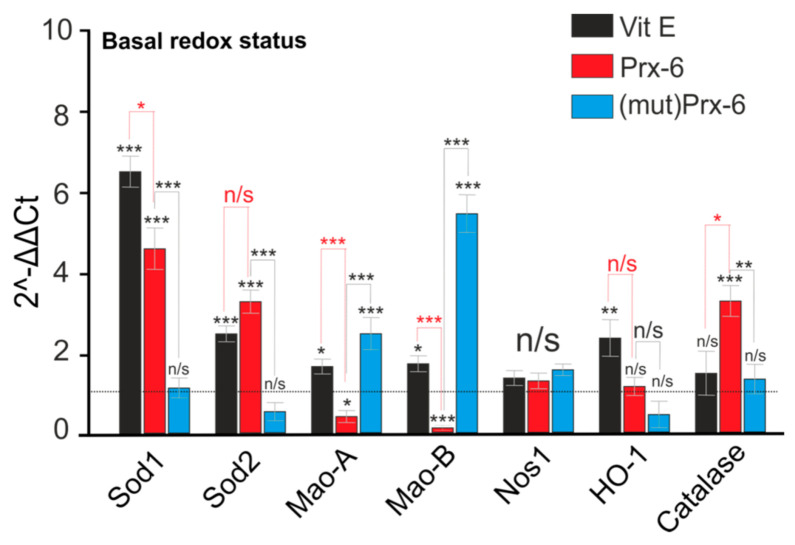
Effect of 24-h incubation with Vit E, Prx-6 and mutPrx-6 on the basal expression of genes involved in regulation of the redox-status. Dashed line level of gene expression in controls (without the impact of the compounds under study). Statistical significance was assessed using paired *t*-test. n/s—data not significant (*p* > 0.05), * *p* < 0.05, ** *p* < 0.01, and *** *p* < 0.001.

Application of exogenous antioxidants or receptors agonists is known to result in changes of genome expression [20,22]. The analysis of the basal expression of genes, encoding regulatory proteins of redox-status cells, showed that a 24-h incubation of hippocampal cells with Vit E leads to a reliable change in the expression of 6 genes out of 7 studied. In particular, there is an increase in expression of genes Moa-A by 99% and Moa-B by 97% (Figure 4), encoding the enzymes monoamine oxidase 1-2 (Moa-A and Moa-B). Vit E, after 24 h of incubation, increased the expression level of genes Sod1 by 621%, Sod2 by 285% and HO-1 by 214%, encoding the enzymes superoxide dismutase 1-2 and Heme oxygenase 1, each is responsible for ROS utilization and can perform protective functions inside the cells. Incubation of cells with Prx-6 leads to an increase in the expression of genes, encoding Sod1, Sod2, and Catalase by 6.3, 3.8, and 3.67 times, respectively (Figure 4). At the same time, the expression of Mao-A and Mao-B decreases. Mutant Prx-6 does not change the expression of most of the studied genes, but the basal level of Mao-A and Mao-B increases 2.4 and 5.8 times (Figure 4).

Thus, the antioxidant enzyme Prx-6 suppresses completely basic and OGD-induced production of mROS and cytROS in hippocampal neurons and inhibits the rate of bursts of ROS production in astrocytes by more than 2-fold that generally leads to improved cell survival rate. In addition, a pronounced effect of Prx-6 on the suppression of NO production in neurons was established. The protective effect of the long-term incubation of the cells with Prx-6 occurs due to regulation of genome expression and changes in the redox-status in the cells. Not only protective properties of Prx-6 are attenuated in the absence of peroxidase activity, but also changes in gene expression occur with predisposition to oxidative stress. The endogenous antioxidant enzyme Prx-6 unlike the exogenous natural antioxidant Vit E exerts a protective effect, whereas Vit E inhibited mROS in neurons under OGD conditions and had no effect on astrocytes.

### 2.3. Antiapoptotic Action of Prx-6 in Ischemia/Reoxygenation

**Figure 5:**
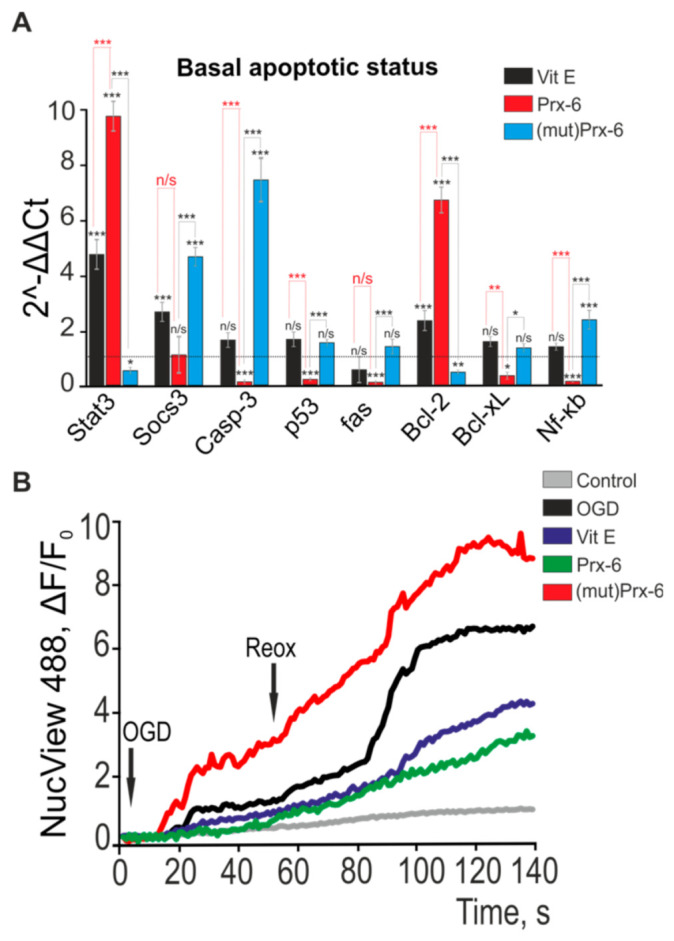
Anti-apoptotic role of 24-h incubation of hippocampal cells with Vit E, Prx-6 and mutPrx-6 under OGD and during reoxygenation. (**A**) Effect of 24-h incubation of hippocampal cells with Vit E (100 μM), Prx-6 (100 μM) and mutPrx-6 (100 μM) on the basal expression of genes involved in regulation of apoptosis. Dashed line level of gene expression in controls (without any effects of the compounds under study). (**B**) Hydrolysis of the fluorogenic substrate of caspase-3 (NucView-488) during a 40-min OGD treatment and the 1.5 h of reoxygenation, indicating apoptosis induction in hippocampal cells. Symbols: Control is the cell culture not exposed to OGD treatment and reoxygenation; OGD denotes cells which were exposed to OGD conditions (40 min) and reoxygenation (1.5 h); Vit E, Prx-6 and (mut) Prx-6 are cells which were incubated for 24 h with the compounds under study exposed to OGD and reoxygenation. Statistical significance was assessed using paired *t*-test. n/s—data not significant (*p* > 0.05), * *p* < 0.05, ** *p* < 0.01 and *** *p* < 0.001.

Analysis of basic expression of genes involved in regulation of apoptosis 24 h after treatment of cells with Prx-6 showed that basal expression of genes encoding protective proteins, Stat3 (signal transducer and activator of transcription 3) and Bcl-2 (Apoptosis regulator Bcl-2), increased by 10 and 6.7 fold, respectively (Figure 5A).Simultaneously, we observed a decrease in expression of the genes encoding pro-apoptotic proteins—Casp-3 (caspase-3) and Fas (Fas cell surface death receptor) by 89%, p53 by 76%, Bcl-xl (B-cell lymphoma-extra large) by 65% and Nf-kb by 85%, which, in general, might indicate an anti-apoptotic effect of Prx-6 on hippocampal cells. The incubation of cells with mutPrx-6 promotes increased expression of pro-apoptotic genes—expression of Socs3 (suppressor of cytokine signaling 3) by 47%, Casp-3 (by 78%) and Nf-kb (by 25.4%); however, the basal gene expression of protective proteins decreased—expression of Stat3 (by 50%) and Bcl-2 (by 60%) (Figure 5A), that might promote activation of cell death process during OGD. The incubation of hippocampal cultures with Vit E is also associated with changes in expression of a number of genes, but these changes are not so pronounced as those observed after incubation of cells with Prx-6. Thus, among the genes of protective proteins there is a 47.3% and 24% increase in expression of Stat3 and Bcl-2, respectively, but, at the same time, a 29.5% increase in expression of pro-apoptotic Socs3 gene is observed (Figure 5A).

Restoration of blood flow in the brain after ischemia (reoxygenation) is even more significant factor that contributes to cell injury via increased ROS production, oxidative stress and activation of apoptosis [8,25]. To identify the impact Prx-6 has on induction of apoptosis under OGD and during reoxygenation (OGD/R), we carried out the experiments, the results of which are shown in Figure 5 and Supplementary Figure S2. Cell cultures were preliminary loaded with the fluorogenic substrate of caspase-3, NucView-488 for an hour. Prior to exposure of cells to OGD, no apoptotic processes were found in the hippocampal culture (due to the absence of NucView-488 fluorescence) (Supplementary Figure S2, Control, middle recording). Then, OGD was created in the cell culture for 40 min, followed by 1.5-h reoxygenation, achieved by replacing the OGD conditions with the oxygen-saturated one (Figure 5B, symbol Reox; Supplementary Figure S2, symbol OGD/R). During the experiment, NucView-488 fluorescence was recorded at a 1 frame interval per 30 s. In the control experiment (Figure 5B, Control), where cells were not exposed to OGD and reoxygenation, a slight NucView-488 fluorescence was observed 2–2.3 h after recording that might be due to the photodestructive effect of the fluorescence excitation on the cells. Activation of caspase-3 and the fluorescence spectra of NucView-488 were recorded at 8 ± 4 min post-OGD-onset and we observed an increase in the fluorescence intensity of the probe that occurred within 40 min of ischemia, and a second peak in caspase-3 activation was seen 25 ± 10 min after reoxygenation (Figure 5B, OGD; Supplementary Figure S2, right recording—Control). The incubation of cells with the antioxidant Vit E for 24 h promotes suppression of caspase-3 activation (Figure 5B, Vit E; Supplementary Figure S2, right recording—Vit E) both under OGD conditions and especially during reoxygenation since the average curve of the fluorescence intensity of NucView-488 is seen below the curve obtained in the stage under OGD conditions. The incubation of cells with Prx-6 also leads to suppression of OGD/R-induced apoptosis, and this antioxidant enzyme inhibits completely the activation of caspase-3 during a 40-min OGD treatment. Only after the onset of reoxygenation the fluorescence intensity of NucView-488 begins to increase (Figure 5B, Prx-6; Supplementary Figure S2, right recording—Prx-6). Mutant mutPrx-6, without peroxidase activity, induces the appearance of the cells with the fluorescence intensity like that of NucView-488 even before OGD/R modeling (approximately 6–12% cells) (Supplementary Figure S2, middle recording-mutPrx-6) and a significant intensification of caspase-3 activation both under OGD conditions and during reoxygenation (Figure 5B, (mutPrx-6); Supplementary Figure S2—mutPrx-6). If we take as 100 the fluorescence intensity of NucView-488 in the experiment carried out under OGD conditions which was recorded after a 40-min OGD treatment and 90 min after reoxygenation, then 24-h incubation of the cultures with mutPrx-6 causes a 58% increase in NucView-488 fluorescence intensity, while the incubation of the same duration with Vit E and Prx-6 leads to a 46% and 53.3% decrease in this parameter, respectively.

Thus, the antioxidant Vit E and the antioxidant enzyme Prx-6 have an anti-apoptotic effect on hippocampal cells via changing basic expression of genes involved in regulation of apoptosis, what is seen through suppression of caspase-3 activity during ischemia and reoxygenation. Although mutant Prx-6 without peroxidase activity loses its ability to suppress apoptosis in the cells, it contributes to this process. This indicates that Prx-6 as an antioxidant enzyme plays a key role in brain cells protection.

### 2.4. The Impact of Prx-6 on Signaling Pathways, Glutamate Receptors and Inflammatory State of Brain Cells

**Figure 6:**
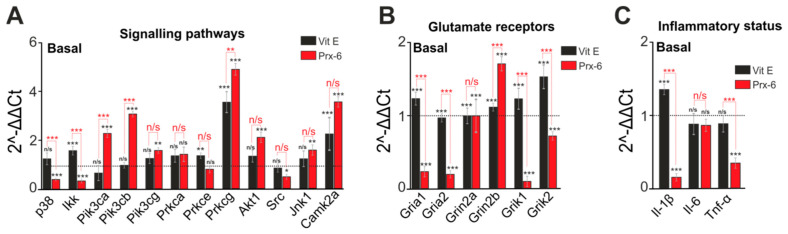
Effect of Prx-6 and Vit E on the basal level expression of genes encoding signal kinases (**A**), subunits of excitatory glutamate receptors (**B**) and proinflammatory factors (**C**). Dashed line level of gene expression in controls (without tested compounds). Statistical significance was assessed using paired *t*-test. n/s—data not significant (*p* > 0.05), * *p* < 0.05, ** *p* < 0.01, and *** *p* < 0.001.

Excitotoxic events, observed during ischemia, may result from changes in the expression of genes encoding subunits of glutamate receptors responsible for excitation of cells as well as signaling kinases associated with regulation of these receptors [26]. A 24-h incubation of hippocampal cells with Prx-6 turned out to lead to significant changes in the basal expression of genes encoding kinases involved in neuroprotective signaling pathways. There is an increase in the expression level of genes encoding subunits of PI3K (phosphoinositide 3-kinase) such as Pik3ca, Pik3cb and Pik3cg by 11.2%, 21.1% and 57%, respectively, as well as a 10.7% increase in the expression level of Akt1 (Akt/protein kinase B) related to PI3K pathway (Figure 6А). Moreover, Prx-6 causes a 42% increase in base expression of the Prkcg gene encoding the isoform of γ protein kinase C (PKC) and an increase in expression of the Camk2a gene encoding calcium/calmodulin-dependent protein kinase II (CaMKII) by 28.6%. When Prx-6 induces an increase in the expression of protective genes, the expression levels of genes encoding proteins which are involved in the regulation of excitotoxicity are significantly suppressed, the expression levels of p38 (p38 mitogen-activated protein kinases), Ikk (IkappaB Kinase) and Src (Proto-oncogene tyrosine-protein kinase Src) decreased by 69%, 72% and 56%, respectively (Figure 6А). Vit E which was used to show differences between the compounds under this investigation, significantly changes expression levels of the considerably smaller number of genes when compared to those under stimulation by Prx-6. There is an increase in the expression of the Prkcg and Camk2a genes by 27.2% and 10.4%, respectively. However, no significant decrease is observed in the expression of genes, encoding proteins, which exert neurotoxic effects, and subjected to Vit E treatment (Figure 6А, black columns).

After 24-h incubation of cells with Prx-6, a decrease in the basal expression of the genes Gria1, Gria2, Grik1 and Grik2, encoding the AMPAR (AMPA-receptor) and KAR (kainate receptor) subunits, by 77%, 81%, 91% and 28%, respectively, was shown. (Figure 6B), which indicates a decrease in the total number of cell membrane receptors. At the same time, an increase in the level of the Grin2b gene encoding NR2B-subunit of NMDAR (NMDA-receptor) is observed, which may be a compensatory mechanism against a decrease in the level of other receptors responsible for excitation of cells. The incubation of the cell cultures with Vit E does not significantly affect the expression of most receptors responsible for excitation of cells, but there is a 53% increase only in the level of Grik2 as compared to control (Figure 6B, red columns). In addition to the effect of Prx-6 on gene expression, it decreases the level of pro-inflammatory genes such as Il-1β (Interleukin-1β) and Tnfα (tumor necrosis factor) by 85% and 66%, respectively (Figure 6C). Vit E did not significantly affect the inflammatory state of hippocampal cells.

Thus, unlike the antioxidant Vit E, Prx-6 showed high efficiency as a regulator in the basal expression of genes encoding proteins responsible for neurotransmission, network excitation and activation of inflammatory processes. Changes in baseline expression of genes under stimulation by Prx-6 are of protective nature, contributing to the suppression of network hyperexcitation, inhibition of inflammatory processes in case of subsequent exposure to ischemia/reoxygenation.

### 2.5. Effect of Prx-6 on OGD/R-Induced Gene Expression

**Figure 7:**
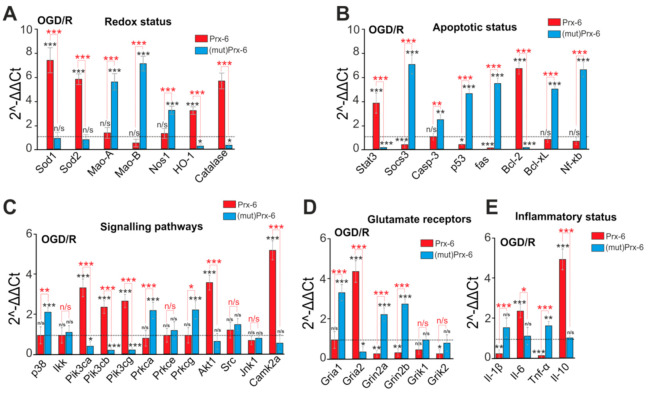
Effect of 24-h incubation of hippocampal cells with Prx-6 (100 μM) and mutPrx-6 (100 μM) on the OGD/R-induced expression of genes involved in regulation of redox status (**A**), apoptotic status (**B**), genes, encoding signalling proteins (**C**), glutamate receptor subunits (**D**) and inflammatory status (**E**). Dashed line level of gene expression in cells after 40 min OGD and 24-h reoxygenation without pre-incubation with Prx-6 (without any effects of the compounds). Statistical significance was assessed using paired *t*-test. n/s—data not significant (*p* > 0.05), * *p* < 0.05, ** *p* < 0.01 and *** *p* < 0.001.

The effect of exogenous Prx-6 persists after 40 min of OGD and 24 h of cell reoxygenation in a CO_2_ incubator. There is an increase in the level of expression of key protective genes—Sod1, Sod2, HO-1 and Catalase by 7.9, 6.4, 3.4 and 5.8 times, respectively (Figure 7A). At the same time, after OGD/R, there is a decrease in the expression level of the Moa-A, Moa-B and Nos genes, and exogenous Prx-6 does not significantly affect their expression. However, mutPrx-6 enhances the course of OGD/R at the level of expression of genes encoding redox status regulator proteins. There is an increase in the expression of Moa-A, Moa-B and Nos1 (Nitric Oxide Synthase 1) by 5.8, 6.7 and 3.7 times, while the level of HO-1 and Catalase decreases (Figure 7A). Prx-6 after OGD/R suppresses the expression of the pro-apoptotic genes Socs3, p53, fas and the expression of anti-apoptotic genes Stat3 and Bcl-2 increases 4 and 6.8 times (Figure 7B). In contrast, mutPrx-6 enhances the expression of pro-apoptotic genes after OGD/R and suppresses anti-apoptotic genes (Figure 7B).

Of the studied genes encoding signaling proteins, after OGD/R, Prx-6 alters the expression of genes encoding PI3K (Pik3c), AKT (Akt1) and CaMKII (Camk2a), which may contribute to post-ischemic cell survival (Figure 7C). mutPrx-6 increased the OGD/R-induced expression of p38 and genes encoding 2 PKC isoforms (Prkca and Prkcg), which, under conditions of glutamate excitotoxicity, may contribute to cell damage.

A decrease in the expression of genes encoding the NMDAR and KAR subunits occurred after incubation of hippocampal cells with Prx-6 and OGD/R, while the expression of the Gria2 gene (GluA2-subunit of AMPAR), on the contrary, increased 4.4 times (Figure 7D). The effect of incubating cells with mutPrx-6 had the exact opposite effect (Figure 7D).

The level of genes encoding pro-inflammatory Il1β and TNFα also decreased in the presence of Prx-6 after OGD/R, and mutPrx-6 led to an increase in the expression of these genes (Figure 7E). Against the background of suppression of the expression of pro-inflammatory genes after incubation of cells with Prx-6 and OGD/R, the gene encoding the anti-inflammatory cytokine, IL-10 (Interleukin-10), is increased by 5.5 times (Figure 7E).

**Figure 8:**
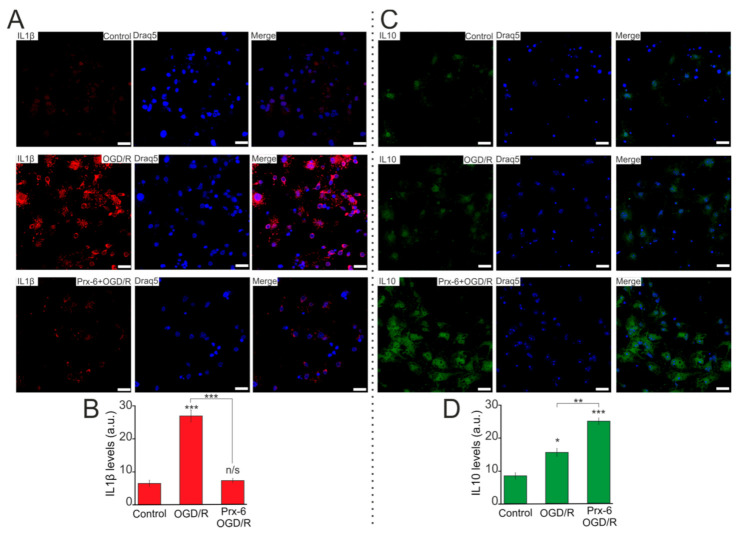
Effect of 24-h incubation of hippocampal cells with Prx-6 (100 μM) on the protein level of IL-1β and IL-10 in Control, after OGD/R and OGD/R with Prx-6. A, C—Immunostaining of IL-1β (**A**) and IL-10 (**C**) of hippocampal cells in the control (without OGD/R), after 40 min OGD (OGD/R) and 24h reoxygenation and 24 h incubation with 100 μM Prx-6, 40 min OGD and 24-h reoxygenation (designated as Prx-6 + OGD/R). Draq5—nuclei staining. (**B**,**D**) Intensity levels of IL-1β and IL-10 were determined by confocal imaging. We analyzed individual cells which had fluorescence of secondary antibodies. The quantative data reflecting the level of IL-1β (**B**) and IL-10 (**D**) expression are presented as fluorescence intensity values in summary bar charts (mean ± SEM). The values were averaged by 150 cells for each column. The results obtained after immunostaining agree well with the data of fluorescent presented in panels **A** and **C**. Each value is the mean ± SE (*n* ≥ 3, *p* < 0.05). Statistical significance was assessed using paired *t*-test. Comparison with OGD/R, *** *p*-level < 0.001, ** *p*-level < 0.01, * *p*-level < 0.05, n/s—data not significant (*p* > 0.05). Comparison Control with Prx6 + OGD/R, * *p*-level < 0.05. Scale bar = 25 µm.

Cell staining with antibodies against IL-1β and IL-10 showed that the level of the protein IL-1β (Figure 8A,B) and IL-10 (Figure 8C,D) increased after OGD/R by 2.4 times and by 24%, respectively. However, in hippocampal cultures after incubation with Prx-6 and OGD/R, there is a 71% decrease in the IL-1β level compared to OGD/R (Figure 8A,B). Whereas the level of IL-10, on the contrary, increases by 57% (Figure 8C,D).

**Figure 9:**
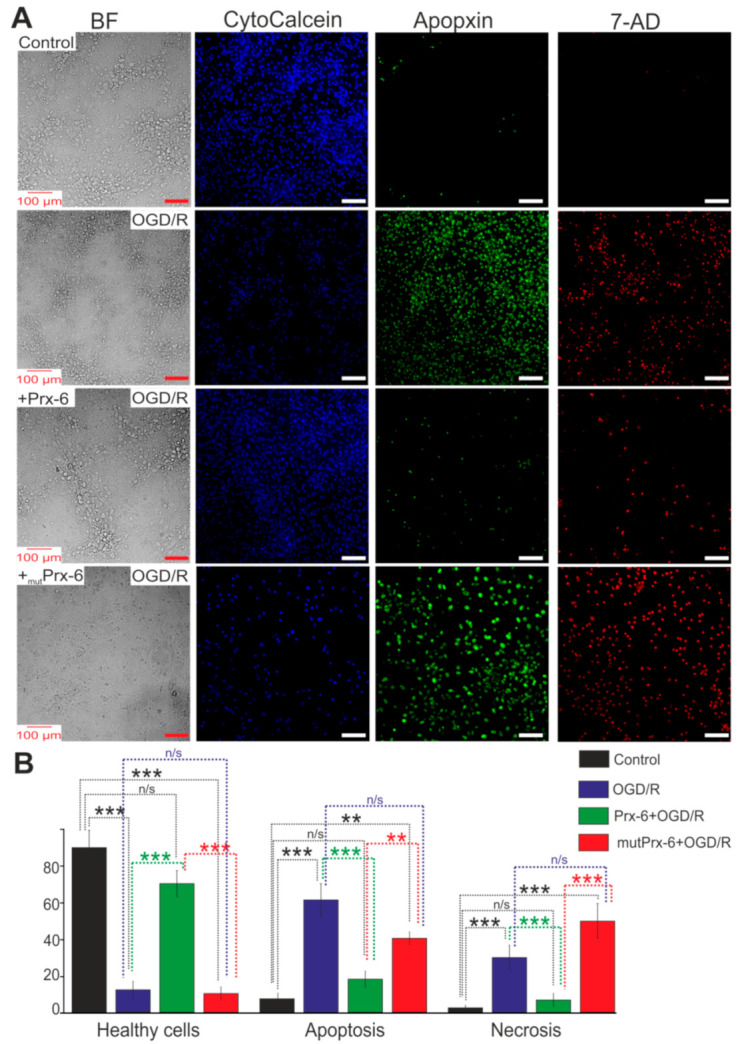
Effect of 24-h incubation of hippocampal cells with Prx-6 (100 μM) and mutPrx-6 (100 μM) on the OGD/R-induced apoptosis and necrosis. (**A**) Cell staining using the Apoptosis/Necrosis Detection Kit assay. BF—bright-field microscopy, CytoCalcein—living cells indicator, Apopxin—apoptotic cells indicator and 7-AD (7-aminoactinomycin D)—necrotic cells indicator. (**B**) Healthy cells and cells with apoptosis or necrosis after 40 min OGD and 24-h reoxygenation. Each value is the mean ± SE (*n* ≥ 4). Statistical significance was assessed using paired *t*-test. n/s—data not significant (*p* > 0.05), ** *p* < 0.01, and *** *p* < 0.001. Scale bar = 100 µm.

At the level of cell survival, all these events with changes in gene expression and suppression of inflammation under the action of Prx-6 lead to a decrease in the percentage of cells with apoptosis and necrosis after OGD/R. The cells were stained using the Apoptosis/Necrosis Detection Kit assay and no more than 8 ± 4% of apoptotic cells and 3 ± 2% of necrotic cells in culture were detected in the control (Figure 9A,B). 24 h after OGD, 61 ± 12% of apoptotic cells and 37 ± 14% of necrotic cells were recorded (Figure 9A,B). Whereas hippocampal cultures incubated for 24 h with Prx-6 survived after OGD/R, and apoptosis and necrosis were detected in 23 ± 7% and 9 ± 5% of cells, respectively (Figure 9A,B). mutPrx-6 did not significantly reduce the number of hippocampal cells killed by OGD/R.

Thus, incubation of cells with Prx-6 leads to an increase in the expression of protective genes also after ischemia-reoxygenation, while mutPrx-6 without peroxidase activity, on the contrary, enhances the expression of genes encoding proteins involved in the activation of damaging signaling pathways. At the same time, after exposure with Prx-6, inflammatory factors were suppressed, which contributed to a decrease in the number of cells dying from necrosis and apoptosis.

## 3. Discussion

The mechanisms of the selective (cell-specific) protective effect of Prx-6 in brain ischemia are poorly understood. For instance, a model of renal ischemia and proteomic analysis were used to show that maximal increase in HSP70 (heat shock protein), PRX3 and PRX6 levels occurred for the first 30 min of ischemia [27]. Prx-6 neutralizes multiple resources of ROS including Н_2_О_2_, peroxynitrite, alkyl peroxides andphospholipid peroxides [28,29]. In our experiments, we demonstrated the protective effect of the antioxidant enzyme Prx-6 on neurons and especially on hippocampal astrocytes [19] while simulating in vitro ischemia. Pre-incubation of the cell cultures with Prx-6 suppresses OGD-induced global increase in [Ca^2+^]_i_, decreases the percentage of the dying cells, promoting suppression of apoptosis under OGD conditions and during reoxygenation via changing baseline expression of the genes encoding proteins which exert a harmful effect of genes encoding protective proteins. The possibility of suppressing Ca^2+^-signaling of the neurons and astrocytes during ischemia or hypoxia by antioxidants taxifolin and Vit E has been shown in our previous studies. At the same time, the role of the antioxidant enzyme Prx-6 in regulation of Ca^2+^-dynamics in neurons and astrocytes is shown for the first time. A significant decrease in expression of genes encoding AMPAR and KAR may contribute significantly to the suppression of OGD-induced Ca^2+^-signals in neurons and especially to the global increase in [Ca^2+^]_i_. The effect of Prx-6 is multiple: it protects both neurons and astrocytes against OGD-induced neurotoxic factors, but its protective effect is most pronounced for astrocytes. This selectivity for astrocytes may be explained by the increased level of Prx-6 expression especially in astrocytes and, hence, by their better adaptation to the protective effect of exogenous additives of Prx-6. Cell-type-specific expression patterns are shown for Prxs isoforms, while Prx-1 and Prx-6 are predominantly expressed in astrocytes and oligodendrocytes and Prx-2-5—in neurons [30,31]. At the same time, the number of astrocytes which express Prx-6 was shown to be 10 times greater than the number of neurons [32]. Therefore, astrocytes might play a key role in the protective properties of Prx-6. It is noteworthy that the expression level of Prx-6 increases in astrocytes and only in some neurons in Parkinson’s disease, Alzheimer’s disease and dementia with Lewy bodies (DLB) [33,34]. This, although indirectly, indicates that Prx-6 tends to have an effect mostly on astrocytes. However, in neurodegenerative diseases, enhanced Prx-6 could not activate the antioxidant defense leading to inevitable depletion of the Prx-6 activity in the cerebral cortex, injury and cell death [16]. Furthermore, it was established that knockout of the gene encoding Prx-6 enhances the sensitivity of tissues and cells to oxidative stress, irrespective of normal level of expression of other antioxidant enzymes [35].

Mitochondria are generators and targets for intracellular ROS at one time. Mitochondrial dysfunctions induced by oxidative stress during ischemia can lead to delayed death of the brain cells [36]. [Ca^2+^]_i_ is the leading intracellular mediator in these processes. An increase in its level contributes to enhancement of ROS production in mitochondria, and its global increase leads to necrotic death of the brain cells during ischemia [4]. Suppression of ROS production with the antioxidant Vit E and the antioxidant enzyme Prx-6 results in decreased number of the cells died from necrosis. Surprisingly, in hippocampal neurons, 24-h incubation with Vit E and Prx-6 led to inhibition of ROS production under OGD conditions, but in astrocytes Vit E had insignificant effect. Given the effects of long-term incubation with different bioactive substances are associated with changes in expression of cell genome, then the differences in the efficiency between Prx-6 and Vit E can be related to their action on genes of the proteins responsible for the redox state.

Our experiments showed that the antioxidants Prx-6 and Vit E suppressed ROS production under OGD conditions due to changes in the expression of genes encoding proteins involved in regulation of the redox state. Moreover, Prx-6 unlike Vit E leads to more pronounced and exclusively positive changes in gene expression. In order to produce energy within the cells, mitochondria generate ROS continuously, and it is necessary to maintain redox balance in the matrix for normal functioning. The lifetime of superoxide anion is 1 ns, because it either dismutates spontaneously or transforms enzymatically by SOD in the mitochondrial matrix. Then, the formed H_2_O_2_ can freely penetrate into the cytosol, performing signaling functions, and its excessive concentrations are normally converted by antioxidant enzymes, including peroxiredoxins [3,37]. The increased gene expression of both SOD2 isoforms after incubation with Prx-6 (versus Vit E) undoubtedly contributes significantly to restriction of ROS production in mitochondrial cells under OGD conditions.

At the same time, the generation of the considerable amount of ROS during ischemia in hippocampal cells, and especially in some populations of GABAergic neurons, takes place in the cytosol [22] due to the activity of NADPH oxidases (NOX). NOX are bound to the cell membrane and generate superoxide into the extracellular space, which is converted into the secondary ROS by enzymatic and non-enzymatic pathways [38]. It is known that NOX2 is also involved in the regulation of pro-inflammatory signaling pathways of TNFα [39]. The expression of the Tnfα gene is also reduced under stimulation by Prx-6 indicating a protective effect of the antioxidant enzyme at the level of the regulation of these proteins. Interestingly, Vit E had no effect on the expression level of TNFα, indicating that these compounds with antioxidant properties have different mechanisms of action.

The pronounced protective effect of Prx-6 on hippocampal cells is supported by the reduced expression of genes encoding Moa-A and Moa-B, which cleave amines, and also are sources of mitochondrial ROS [40]. Numerous studies report that Moa-A is expressed mainly in neurons, and Moa-B—in astrocytes [41,42]. Moreover, it is established that inhibitors of Moa-B prevent the formation of the dopamine-induced mPTP [43]. In our experiments, Prx-6 suppressed both monoamine oxidase isoforms, but Moa-B exerted more pronounced effect that might also contribute to better protection of astrocytes under OGD conditions. Other mechanisms underlying ROS suppression in hippocampal mitochondria under stimulation by Prx-6 are also possible. For example, in the cell line of THP-1 cells characterized by ROS overproduction, it has been found that due to the binding to the C-terminal domain of TRAF6 protein, endogenous Prx-6 inhibited the formation of TRAF6-ECSIT complex, thereby reducing ROS formation in mitochondria [44].

The mutant Prx-6 without antioxidant activity enhances the formation of mROS under OGD, contributing equally to the death of both astrocytes and neurons, probably due to negative effects on the expression of genes encoding redox status proteins. The mutPrx-6 enhances the expression of Nox2 and Nox4, Moa-A and Moa-B, inhibiting in this case protective Sod2 and HO-1. Heme oxygenase-1 (HO-1) is an inducible heme oxygenase and is normally expressed at low levels in the brain and only in certain groups of neurons and astrocytes. An increased expression of HO-1 occurs under the action of several oxidative stimuli, promoting cell protection through antioxidant and anti-inflammatory responses [45,46]. A decrease in HO-1 expression after incubation with mutPrx-6 may lead to suppression of the antioxidant capacity of the brain cells.

Oxidative stress that occurs under OGD conditions is linked to induction of not only necrosis, but also apoptosis. Reoxygenation that follows after OGD can enhance the activation of apoptosis in hippocampal cells [20]. At the same time, mRNA level of Prx-6 is known to increase significantly not only after oxidative stress, but also during reoxygenation [47]. In our experiments it has been established that 24-h incubation of hippocampal cells with Prx-6 increases the anti-apoptotic status via changing the expression level of key genes resulting in inhibition of Caspase-3 under OGD and especially during reoxygenation. Nuclear factor-κB (NF-κB) is one of the key proteins in the regulation of apoptosis; the baseline level of expression of the gene encoding NF-κB is almost completely suppressed after incubation with Prx-6. NF-κB transcription factor is activated in response to oxidative stress and can activate both pro- and anti-apoptotic signaling pathways [48]. It is commonly known fact that Prx-6 expression is regulated by many transcription factors, for instance, erythroid 2-related factor 2 (NRF2), hypoxia-inducible factor 1-alpha (HIF1α) and CCAAT-enhancer-binding protein-β (C/EBPβ), they lead to increased Prx-6 levels, and NF-κB, in its turn, suppresses the expression of this protein [49]. Our study, treatment of the cells with exogenous Prx-6 for 24 h leads to suppression of the expression level of the gene encoding NF-κB, that may have a neuroprotective effect and be a feedback mechanism promoting the production of endogenous Prx-6. It is known that some antioxidants such as aspirin, sodium salicylate, indometacine, L-cysteine, N-acetylcysteine and also vitamin Е are capable of suppressing the activity of NF-κB [50,51], but in our experiments only Prx-6 exerted antioxidant properties. Furthermore, kidney cells were used to show that the expression level of the genes of transcription factor AP-1 and caspase-3 increased after I/R injury by 6 and 5 fold, respectively, inducing cell death through an apoptotic pathway. The level of expression of AP-1 after I/R injury in the group of animals pretreated with Prx-6 increased only by 3 fold, and the expression of caspase-3 was even 2 times lower than that in control animals (in the absence of I/R injury) [52]. Similarly, in our experiments on hippocampal cells, the expression of caspase-3, p53 and Fas was significantly decreased after 24-h incubation with Prx-6, while the mutPrx-6 increased the expression of these genes and favored apoptosis. The protective effect of Prx-2 is shown through inhibition of p53-Bax-mediated death pathway [53]. In a similar manner in our experiments, Prx-6 through inhibition of baseline expression of p53, improved the survival rate of hippocampal cells under OGD. The regulation of signaling kinases expression may also be involved in the realization of neuroprotective effects of Prx-6. In addition to antioxidant action, Prx-6 has been shown to have an important signaling function through regulation of Ca^2+^-independent phospholipase A2 (aiPLA2) activity, which is widely expressed in acidic intracellular pools [54,55]. Prx-6 stimulates signaling pathways of p38 and PI3K/Akt thereby promoting the formation of arachidonic acid and regulation of the growth and division of the cells via Src (SFK) kinases. In our experiments, treatment with Prx-6 leads to enhanced expression of genes encoding neuroprotective kinases PI3K and Akt, and taking into account that this enzyme also suppresses the expression of NF-kB and TNFα, we may conclude that anti-apoptotic action of Prx-6 is mediated via complex signaling cascade. The activation of NF-kB via IKK kinase is known to be a common event for TNFα and PI3K-Akt signaling pathways [56], and the expression level of Ikk and NF-kB decreases under stimulation by Prx-6. The participation of PI3K-Akt cascade in antiapoptotic action of Prx-6 is supported by the decreased expression of p53 and Fas genes, probably due to activation of the above said signaling cascade. It has been reported that Akt-induced decrease in expression of p53 contributes to the survival of hippocampal neurons in hypoxia [57]. The involvement of JNK/AP-1 signaling cascade [58] in the activation of apoptosis is also known and inhibition of these proteins by antioxidants prevents cell death when H_2_O_2_ is added [59]. Exogenous Prx-6, through a decrease in ROS concentration, prevents AP-1 activation, suppressing apoptosis [24]. In our experiments, incubation with Prx-6 tended to increase gene expression of JNK-signaling cascade that was shown for the first time on brain cells. Moreover, increased baseline expression of Camk2a gene encoding CaMKII after incubation with Prx-6 may also exert a protective effect on brain cells. We have previously shown that CaMKII deficiency is a contributing factor of the injury of mechanisms underlying hypoxic preconditioning, while activation of an alternative signaling pathway of PI3K restores this mechanism of neuroplasticity [60].

Restoration of blood flow after stroke and reoxygenation of the brain with thrombolytics is a widely used approach for the treatment of ischemic stroke. On the other hand, reperfusion therapy has a short time limit and the use of this approach beyond this optimal time can lead to blood–brain barrier damage, hemorrhagic transformation and massive brain edema [61]. Neuroinflammation contributes substantially to brain tissue injury during the primary and secondary progression of brain ischemia/reperfusion injury [62]. Moreover, anti-inflammatory drugs and anti-intracellular cell adhesion molecule 1 (ICAM-1) antibody (enlimomab) and cyclooxygenase inhibitors (indomethacin and paracetamol) did not show outstanding results in ischemic stroke in clinical trials [63]. One of the promising approaches in the prevention and treatment of I/R injuries is the usage of antioxidant enzymes, which are more effective than low-molecular-weight antioxidants of both natural and synthetic origin [64]. Indeed, in our experiments, Prx-6 not only suppressed necrotic cell death after a 40-min OGD treatment, but also showed greater efficacy in reducing the baseline expression of genes encoding pro-inflammatory factors IL-1β and TNFα as compared to that after addition of Vit E. A decrease in TNFα expression after incubation with Prx-6 may be associated with astrocyte activation [65], which of course requires further study.

Post-ischemic neuro-inflammation also occurs during activation of Toll-like receptors (TLR), which belong to the family of highly conserved innate immunity receptors that recognize DAMPs that are released from ischemic brain tissue [66,67]. It is shown, that ischemia simultaneously increases the expression of Toll-like receptor-4 (TLR4) and extracellular release of ligands for this receptor from damaged nerve cells, which triggers inflammatory signaling cascades in the remaining cell populations [66,68]. Proteins of the peroxiredoxin family are released into the extracellular space 12–24 h after the onset of stroke, and Prx-5 and Prx-6 are able to activate macrophages through the TLR4-dependent signaling cascade [69]. The ligustilide compound (3-butylidene-4,5-dihydrophthalide) has a protective effect via inhibition of the TLR4/Prx-6 signaling pathway and suppression of neuroinflammation [32]. In our experiments, the addition of exogenous Prx-6 leads exclusively to anti-inflammatory effects, recorded by a decrease in the expression level of Il1-β and Tnfα, which did not occur after incubation with Vit E.

## 4. Materials and Methods

Experimental protocols were approved by the Bioethics Committee of the Institute of Cell Biophysics. Experiments were carried out according to Act708n (23 August 2010) of the Russian Federation National Ministry of Public Health, which states the rules of laboratory practice for the care and use of laboratory animals, and the Council Directive 2010/63 EU of the European Parliament on the protection of animals used for scientific purposes.

### 4.1. Preparation of Mixed Hippocampal Neuroglial Cell Cultures

Cell cultures were prepared as described in detail previously [8]. Briefly, 0–1 day old pups were euthanized and decapitated. The extracted hippocampus was washed with Mg^2+^- and Ca^2+^-free Versene solution and minced with scissors. Then, the tissue fragments were digested with 1% trypsin solution for 10 min at 37 °C and washed two times with cold Neurobasal-A medium. Trypsinized tissue was gently triturated with a pipette, and the debris was then carefully removed with a pipette tip. The obtained cell suspension was seeded on polyethyleneimine-coated glass coverslips and grew for 10–12 days in vitro in the cell culture medium composed of Neurobasal-A medium, supplement B-27 (2%) and 0.5 mM glutamine.

The drugs were added into culture medium under sterile conditions in the case of experiments with 24-h pre-incubation with Prx-6 (Human recombinant PRDX6, BioVision, CA, USA), mutPrx-6 (mutant variant Prdx6-C47S, kindly provided by professor V.I. Novoselov) [24] and Vit E (α-Tocopherol, Sigma-Aldrich, St. Louis, MO, USA). Then, the cell cultures were washed after the pre-incubation with Hank′s balanced salt solution and used in experiments.

### 4.2. Immunocytochemical Method

In order to detect GFAP, NeuN, IL-1β or IL-10 in cells, we used an immunocytochemical assay. The cells were fixed with 4% paraformaldehyde +0.25 % glutaraldehyde in PBS for 20 min and washed three times with ice-cold PBS for 5 min. Glutaraldehyde was added into the fixative solution to minimize washing of antibodies from cells during permeabilization. To permeabilize cells, we used 0.1 % Triton X-100 solution for 15 min. Fixed cells were incubated in 10% donkey serum for 30 min at room temperature to block non-specific antibody binding sites. The cells were then incubated with primary antibodies against investigated proteins for 12 h at 4 °C. The fixed cells were subsequently washed with PBS (3 times for 5 min) and probed with secondary antibodies conjugated with fluorescent label manual. We used purified rabbit polyclonal Anti-NeuN antibody (Abcam, RRID: AB_10711153), purified mouse monoclonal anti-GFAP antibody (BioLegend, RRID: AB_2632644), purified monoclonal armenian hamster monoclonal IL-1β antibody (B122, sc-12742), rabbit polyclonal anti-IL10 antibody (SAB5700775, Sigma-Aldrich), donkey polyclonal secondary antibody to rabbit IgG (H + L) (Alexa Fluor-647) (Jackson ImmunoResearch Europe LTD, RRID: AB_2492288), donkey polyclonal secondary antibody to mouse IgG—H&L (Alexa Fluor-594) (Abcam, RRID: AB_2732073). Dilutions of primary and secondary antibodies were performed according to the manufacturer’s recommendations for immunocytochemical staining. The fluorescence of antibodies was visualized with an inverted confocal microscope Leica TCS SP5 (Leica, Wetzlar, Germany). Registration of the secondary antibodies fluorescence for the control and experimental groups of cell cultures was carried out at the same microscope setting. Fluorescence analysis was performed in ImageJ 2002 software (RRID: SCR_003070) using the Analyze particles and Time series analyzer plugins.

### 4.3. Fluorescent Ca^2+^ Measurements

To detect the changes in [Ca^2+^]_i_, hippocampal cell cultures were loaded with Fura-2 (4 µM; 40 min incubation; 37 °C). The cells were stained with the probe dissolved in Hank‘s balanced salt solution (HBSS) composed of (mM): 156 NaCl, 3 KCl, 2 MgSO_4_, 1.25 KH_2_PO_4_, 2 CaCl_2_, 10 glucose, and 10 HEPES, pH 7.4. To measure [Ca^2+^]_i_, we used the system based on an inverted motorized microscope Leica DMI6000B with a high-speed monochrome CCD-camera HAMAMATSU C9100. For excitation and registration of Fura-2 fluorescence, we used FU-2 filter set (Leica, Germany) with excitation filters BP340/30 and BP387/15, beam splitter FT-410, and emission filter BP510/84. Illuminator Leica EL6000 with a high-pressure mercury lamp was used as a source of excitation light. To distinguish neurons and astrocytes, we used short-term applications of 35 mM KCl and 10 µM ATP before the main experiments. This method was described in detail in our previous work [70]. Briefly, KCl induces depolarization of excitable cells, which contain a wide range of voltage-gated cation channels. KCl-induced depolarization promotes the opening of voltage-gated calcium channels in neurons (predominantly L-type channels). The conductivity and density of cation channels in astrocytes are insufficient to evoke high-amplitude Ca^2+^ response to KCl application. All the Ca^2+^ signals are presented as 340/380 ratio of Fura-2 fluorescence.

### 4.4. Fluorescent ROS Measurements

For recordings of changes in mitochondrial or cytosolic ROS production, hippocampal cell cultures were loaded with MitoSOX Red (mitochondrial ROS indicator, 5 µM, 15 min incubation; 37 °C) or H_2_DCF-DA (mainly cytosolic ROS indicator; 10 µM, 20 min incubation; 37 °C). After incubation with the dyes, cells were washed three times before the experiment. To measure the ROS generation, we used the system based on the inverted motorized microscope Leica DMI6000B with a high-speed monochrome CCD-camera HAMAMATSU C9100 and a high-speed light filter replacing system Leica’s Ultra-Fast Filter Wheels with replacing time 10–30 ms. For excitation of DCFH_2_-DA and MytoSOX Red we used L5 filter set (Leica, Germany) with excitation filter BP480/40, dichroic mirror 505 and emission filter 527/30. We determined the shape ROS production rates under oxygen-glucose deprivation (OGD).

### 4.5. Fluorescent NO and [Ca ^2+^]_i_ Measurements

NO production was evaluated using the fluorescent probe DAF-FM diacetate (Molecular Probes, Eugene, OR, USA), oxidation of which by NO dramatically increases the quantum yield of the dye. Cells were loaded with 5 μM DAF-FM for 40 min at 37 °C. For simultaneous monitoring of intracellular NO and Ca^2+^, 4μM Fura-2AM was added to the medium. After loading, the cells were additionally incubated in Hanks balanced salt solution (HBSS) for 20 min to complete the deesterification of the dyes. Dye-loaded cells were visualized using a Cell Observer imaging system (Carl Zeiss, Oberkochen, Germany). DAF-FM fluorescence was excited using a BP 475/40 filter. BP 340/30 and BP 387/15 filters were used to excite the Ca^2+^-bound and Ca^2+^-free forms of Fura-2AM, respectively. The emission of both DAF-FM and Fura-2 was recorded at 530 ± 25 nm. In the case of double staining (Fura-2AM and DAF-FM), the total exposure time per three-channel frame was 20 s. Collected 8-bit time-lapse images were analyzed using the ImageJ software with Time Series Analyzer and RatioPlus plugins. The experimentally obtained curves for NO were smoothed to decrease the effect of noises. The curve ΔF/F_0_ characterizes the fluorescence intensity of benzotriazole, a product of nitrosylation of DAF-FM accumulated in the cells.

### 4.6. The Technique for Simulation of Ischemia-Like Conditions

Ischemia-like conditions (oxygen-glucose deprivation, OGD) were obtained by omitting glucose (HBSS medium without glucose) and by displacement of dissolved oxygen with argon in the leak-proof system [20]. The level of oxygen in the medium was measured using a Clark electrode. Oxygen tensions reached values 30–40 mm Hg or less within 20 min after the beginning of displacement. Ischemia-like conditions lasting for 40 min were created using supplying the oxygen-glucose deprivation (OGD)-medium into the chamber with cultured hippocampal cells. Constant argon feed into the experimental chamber was used to prevent the contact of the OGD-medium with the atmospheric air.

### 4.7. Assessment of Cell Viability and Apoptosis

Propidium iodide (1 µM) were used to evaluate the number of dead cells in the cell cultures before and after OGD. The cells were stained for 5 min with the probes diluted in HBSS and then rinsed with HBSS. Fluorescence of the probes was detected with an inverted fluorescent microscope Zeiss Axio Observer Z1 using Filter Set 20. Cell death induced by OGD was assessed by propidium iodide staining (PI, 1 µM) before and after the exposures in the same microscopic field. Since PI stains both dead astrocytes and neurons, analysis of calcium signals upon 35 mM KCl application before OGD was used to identify the type of cells. Neurons were identified by the fast transient calcium signal upon KCl application as described previously. Furthermore, we used the Ca^2+^ signals (presence or absence of a global increase in [Ca^2+^]_i_ during OGD) as an additional indicator of cell viability [19].

To simultaneously monitor apoptotic, necrotic and healthy cells after Prx-6 treatment and 24 h OGD/R with fluorescence microscope, Apoptosis/Necrosis Detection Kit was used. Cells were washed 1–2 times and resuspended with Assay Buffer. To detect apoptotic cells, Apopxin Green Indicator was used. Apoptotic cells were visualized using the FITC channel (Ex/Em = 490/525 nm). For staining necrotic cells, we used 7-aminoactinomycin D (Ex/Em = 550/650 nm). To detect healthy cells, CytoCalcein 450 was used and cells were visualized using the violet channel (Ex/Em = 405/450 nm).

To examine the effect of antioxidants on OGD-induced initiation of apoptosis, a fluorescent probe, NucView488 caspase-3 substrate, was used for staining. In this regard, before the experiments, the cultures were loaded with NucView488 for 1 h (final concentration of 2 μm). The cultures were then subjected to 40-min oxygen-glucose deprivation and 2-h reoxygenation. NucView488 fluorescence was recorded using an image analysis system based on an Axiovert 200M inverted fluorescence microscope equipped with a Hamamatsu ORCA-Flash 2.8 high-speed monochrome CCD camera. A Lambda DG-4 Plus illuminator (Sutter Instruments, Novato, CA, USA) with a high-pressure mercury lamp was used. To excite and register the NucView488 emission, we used a set of light filters: Filter Set 10 with excitation filter BP 450–490, beam splitter FT510, emission filter BP 515–565. Five independent regions of each glass with cell culture were analyzed. Each experimental series consisted of at least three separate repeats. All the results are presented as mean ± standard error.

### 4.8. Extraction of RNA

Mag Jet RNA Kit (Thermo Fisher Scientific, Waltham, MA, USA) was used for the extraction of total RNA. The RNA quality was estimated by electrophoresis in the presence of 1 μg/mL ethidium bromide (2% agarose gel in Tris/Borate/EDTA buffer). The concentration of the extracted RNA was determined with NanoDrop 1000c spectrophotometer. RevertAid H Minus First Strand cDNA Synthesis Kit (Thermo Fisher Scientific, Waltham, MA, USA) was used for reverse transcription of total RNA.

### 4.9. Real-Time Polymerase Chain Reaction (RT-qPCR)

Each PCR was performed in a 25 μL mixture composed of 5 μL of qPCRmix-HS SYBR (Evrogen, Moscow, Russia), 1 μL (0.2 μM) of the primer solution, 17 μL water (RNase-free), 1 μL cDNA. Dtlite Real-Time PCR System (DNA-technology, Moscow, Russia) was used for amplification. Amplification process consisted of the initial 5 min denaturation at 95 °C, 40 cycles of 30 s denaturation at 95 °C, 20 s annealing at 60–62 °C, and 20 s extension step at 72 °C. The final extension was performed for 10 min at 72 °C. All the sequences were designed with FAST PCR 5.4 and NCBI Primer-BLAST software. The data were analyzed with Dtlite software (DNA-technology, Moscow, Russia). The expression of the studied genes was normalized to gene encoding Glyceraldehyde 3-phosphate dehydrogenase (GAPDH). Data were analyzed using Livak′s method [71].

### 4.10. Statistical Analysis

All presented data were obtained from at least three cell cultures from 2–3 different passages. All values are given as mean ± standard error (SEM). Statistical analyses were performed by Two-way ANOVA, followed by Sidak’s multiple comparison test or by paired *t*-test. Differences are significant * *p* < 0.05, ** *p* < 0.01, and *** *p* < 0.001. n/s—data not significant (*p* > 0.05). MS Excel, ImageJ, Origin 2016 (OriginLab, Northampton, MA, USA), and Prism GraphPad 7 (GraphPad Software, RRID: SCR_002798) software was used for data and statistical analysis.

## 5. Conclusions

Thus, exogenous Prx-6 has a complex protective effect on hippocampal cells during ischemia with tendency towards more selective protection of astrocytes. Incubation with Prx-6 changes the basic antioxidant status of cells through changes in the expression of key genes, resulting in the suppression of ROS production by mitochondria under OGD conditions. The anti-apoptotic effect of Prx-6 also occurs through an increase in the expression of genes encoding protective kinases and transcription factors while suppressing proteins with toxic effects. The protective effect of Prx-6 also includes its anti-inflammatory effect and suppression of the expression of glutamate receptors involved in the excitation of cells, thereby contributing to inhibition of global increase in [Ca^2+^]_i_ and necrosis.

## Data Availability

The data presented in this study are available on request from the corresponding author.

## Supplementary Materials

The following are available online at https://www.mdpi.com/article/10.3390/ijms22168805/s1.
