# Supplementary material for: Mechanisms Underlying the Protective Effect of the Peroxiredoxin-6 Are Mediated via the Protection of Astrocytes during Ischemia/Reoxygenation
Source: Int J Mol Sci. 2021 Aug 16;22(16):8805. doi: 10.3390/ijms22168805 (PMC8396200; doi:10.3390/ijms22168805)
Supplement: Supplementary file 1 [file ijms-22-08805-s001.zip › ijms-1304213-supplementary.pdf]

Supplementary Materials

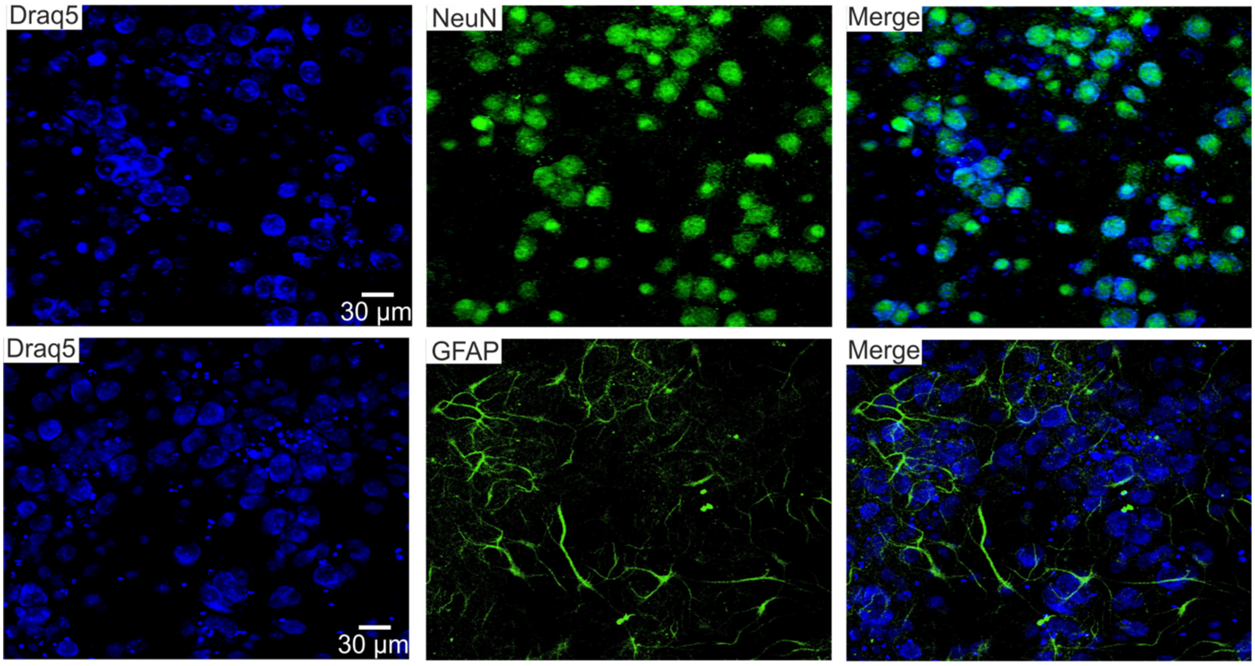

Figure S1. Immunocytochemical staining of hippocampal cell culture (10 DIV) with astrocytic marker, antibodies against glial fibrillary acidic protein (GFAP) and neuronal marker (NeuN). The nuclei of all cells stained with Draq5 are shown in blue.

A

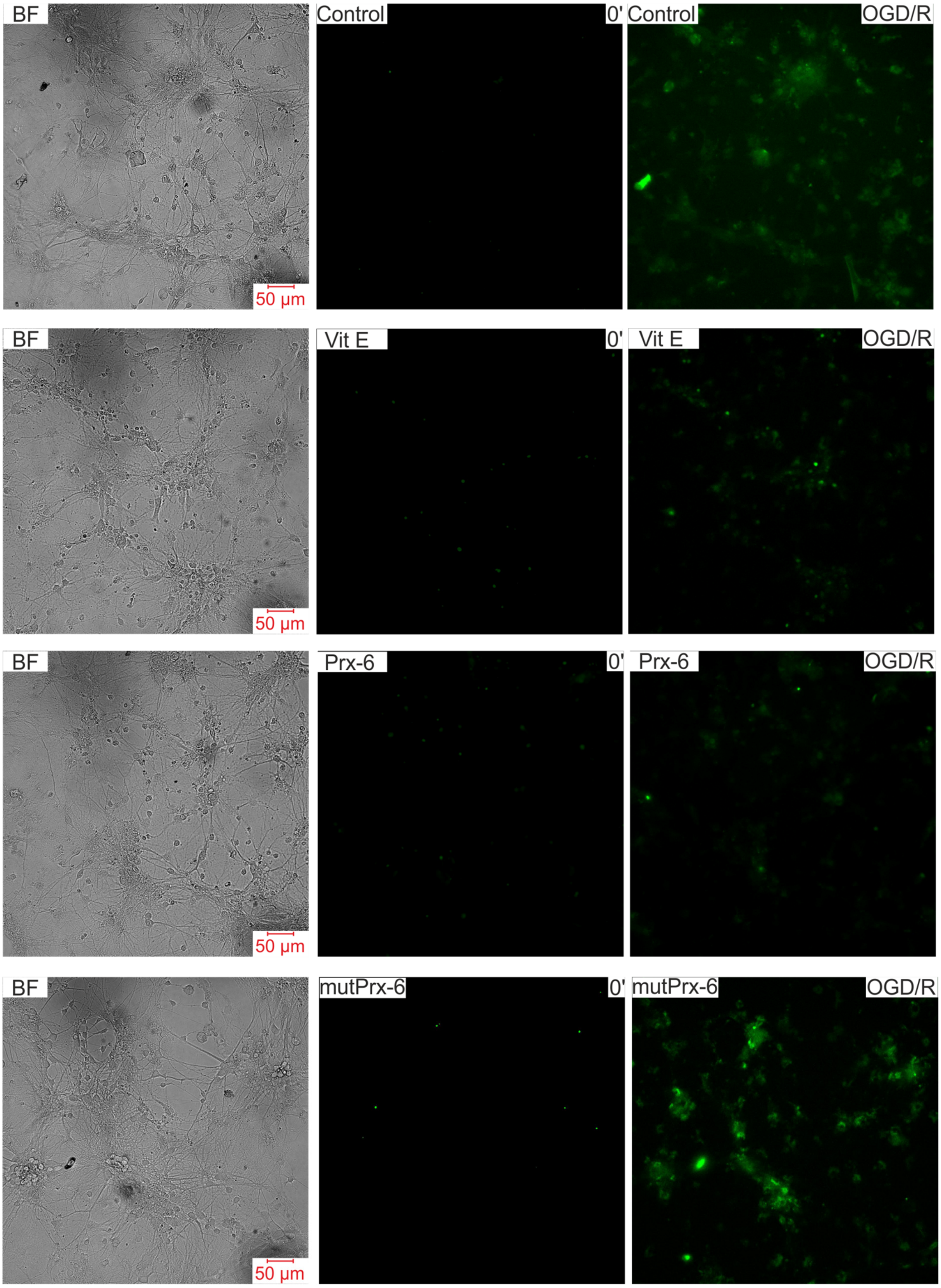

Figure S2. Images of hippocampal cell cultures loaded with a fluorogenic substrate of caspase-3, NucView-488, which were exposed to OGD conditions and reoxygenation. The appearance of green fluorescence after OGD/R indicates the induction of apoptosis in hippocampal cells. Images of the cell culture in transmitted light (BF), NucView-488 fluorescence detection channel before experiments (0'), and after 40-min OGD treatment and the 1.5 h of reoxygenation (OGD/R) are represented. Images correspond to the curves shown in Figure 5B.
